# The Hepatoprotective Effect of Piperine Against Thioacetamide-Induced Liver Fibrosis in Mice: The Involvement of miR-17 and TGF-β/Smads Pathways

**DOI:** 10.3389/fmolb.2021.754098

**Published:** 2021-10-29

**Authors:** Amr M. Abdelhamid, Ayman Selim, Mai A. Zaafan

**Affiliations:** ^1^ Biochemistry Department, Faculty of Pharmacy, October University for Modern Sciences and Arts (MSA), 6th of October City, Egypt; ^2^ Faculty of Pharmacy, October University for Modern Sciences and Arts (MSA), 6th of October City, Egypt; ^3^ Pharmacology and Toxicology Department, Faculty of Pharmacy, October University for Modern Sciences and Arts (MSA), 6th of October City, Egypt

**Keywords:** piperine, liver fibrosis, smad-3, TGF-β, mice 3

## Abstract

Liver fibrosis is characterized by a series of events including activation of quiescent hepatic stellate cells (HSCs) into proinflammatory, contractile, and fibrogenic myofibroblasts, which is the primary trigger for the fibrogenesis process. HSC activation involves many signaling pathways such as the TGF-β/smads pathway. Specific microRNAs have been identified to play a crucial role in the activation of HSCs *via* various signaling pathways. Piperine has recently been studied as a promising anti-fibrotic agent against pancreatic fibrosis through altering the TGF-β1/Smad pathway. Hence, the current study evaluated the beneficial effects of piperine in thioacetamide-induced liver fibrosis in mice through the modulation of miRNA-17 and TGF-β/smads pathways. Mice were allocated into three groups randomly. Thioacetamide was used to induce liver fibrosis for 6 weeks. Starting from the fourth week of the experiment, mice were treated with piperine daily for 21 days. Piperine treatment resulted in a significant downregulation of miRNA-17 expression, leading to the restoration of smad-7 accompanied with marked inhibition of TGF-β/smads signaling with further suppression of the activated HSCs and collagen deposition in the hepatocytes. In conclusion, piperine has the potential to be a promising therapeutic drug for the treatment of liver fibrosis through inhibiting the TGF-β/smads pathway.

## Introduction

Hepatic fibrosis is a long-term and consequent event of chronic liver injury and inflammation (hepatitis). Chronic liver disease affects more than 800 million people worldwide, and it results in 2 million deaths yearly (Parola and Pinzani, 2019). Hepatic fibrosis can progress into cirrhosis which usually develops into liver cancer, mostly HCC, the seventh most frequently occurring cancer and the second most fatal cancer worldwide (McGlynn et al., 2021). Liver injury can be induced by hepatotropic viruses, drug toxicity, alcohol, lipocyte deposition (steatosis), aflatoxin B_1_, and tobacco (Boyer and Lindor, 2016; McGlynn et al., 2021). Prolonged exposure to the etiologic factors leads to the development of chronic inflammation of the liver, which is expressed by necrosis and apoptosis of hepatocytes. These processes induce a series of events including activation of quiescent hepatic stellate cells (HSCs) into proinflammatory, contractile, and fibrogenic myofibroblasts, which is the primary trigger for the fibrogenesis process (Bataller and Brenner, 2005; Boyer and Lindor, 2016).

**GRAPHICAL ABSTRACT:**
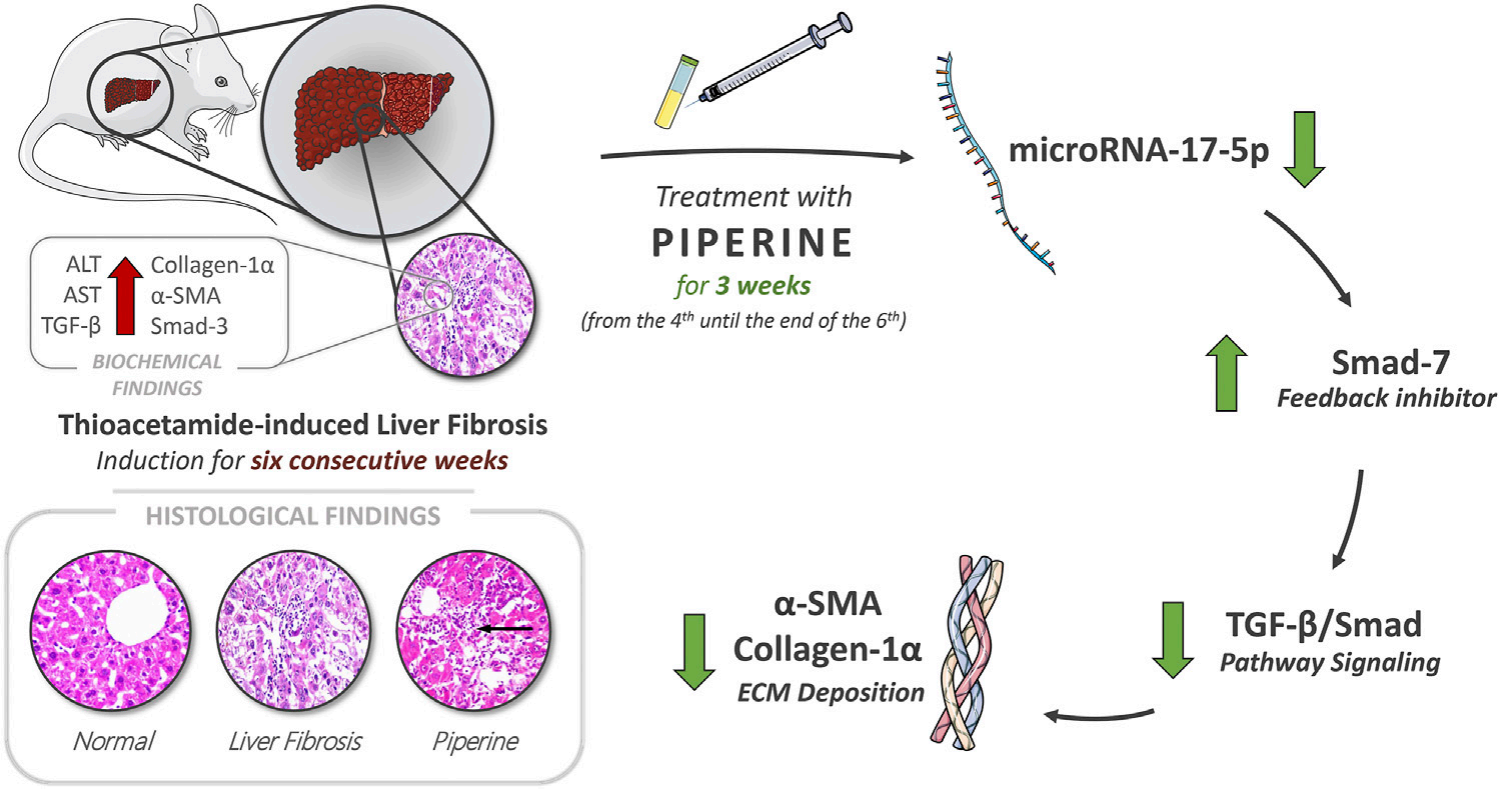
Piperine possesses a potential therapeutic effect on hepatic fibrosis by restoration of liver enzymes, AST and ALT levels, and through targeting the TGF/Smad signaling pathway resembled in the decrease in expression levels of TGF-β, Smad-3, and miR-17-5p and the increase in the expression level of the negative feedback inhibitor Smad-7, in addition to regression of ECM accumulation by reducing α-SMA deposition and collagen-α1 relative expression.

Fibrogenesis is expressed as an imbalance between the process of deposition and degradation of extracellular matrix (ECM) proteins by the tissue inhibitor of metalloproteinase (TIMP) and degrading enzyme matrix metalloproteinase (MMP). This imbalance aggravates the excess accumulation of the altered composition of ECM proteins including fibrillar collagen type I and III, alpha smooth muscle actin (α-SMA), non-muscle myosin, fibronectin, and vimentin, which lead to scar tissue formation, eventually (Bataller and Brenner, 2005; Boyer and Lindor, 2016).

The hepatic fibrogenesis process is orchestrated by several profibrogenic cytokines. TGF-β1 is the most important cytokine because of its pivotal role in several pathophysiologic processes. It is responsible for the activation of myofibroblasts and induction of ECM production through activation of the standard TGF-β1/Smad signaling pathway and non-standard pathways such as mitogen-activated protein kinase (MAPK) and mammalian target of rapamycin (mTOR) (Meng et al., 2016; Dewidar et al., 2019).

The TGF- β1/Smad signaling pathway is initiated by the release of activated TGF-β1 in order to bind to TGF-β1 receptor type II (TβRII). After, TβRII induces recruitment of TβRI and its phosphorylation in order to sensitize it for its substrates Smad2 and Smad3. Smad2 and Smad3 are phosphorylated by TβRI producing pSmad2 and pSmad3, which can complex directly with Smad4. The formed complex can enter the nucleus and has the affinity to bind to the DNA, and consequently, it intervenes in the transcription of multiple gene targets which are responsible for the production of many profibrotic molecules including alpha-smooth muscle actin (α-SMA), collagen I, TIMP, and connective tissue growth factor (CTGF). Meanwhile, Smad7 possesses an inhibitory role in the latter phosphorylation by competing with Smad2 and Smad3 binding to TβRI; accordingly, it downregulates the signaling, thus acting as the negative feedback inhibitor (Meng et al., 2016; Hu et al., 2018; Dewidar et al., 2019).

MicroRNAs (miRNAs) are short, 18- to 24-base, noncoding single-stranded RNAs with several gene-expression modulatory activities. They regulate protein translation by partial or complete binding to targeted mRNA, resulting in its degradation or inhibition of binding to rRNA; thus, they act as post-transcriptional gene modulators (Shaker et al., 2020) (Shaker et al., 2021). miRNAs have been extensively investigated in the past years due to their various important roles found in liver fibrosis, either by pro-inflammatory or anti-inflammatory activities (He et al., 2012; Boyer and Lindor, 2016; Zhao et al., 2019; Zaafan and Abdelhamid, 2021). miR-17-5p is an example of pro-inflammatory miRNA involved in fibrosis *via* manipulation of several pathways such as repression of the inhibitory Smad-7 signaling pathway, which results in termination of the negative feedback inhibition to the TGF-β1/Smad pathway (Yu et al., 2015; Zhang et al., 2018; Wang et al., 2019; Fu et al., 2021), the peroxisome proliferator-activated receptor alpha (PPAR-α) pathway (Du et al., 2015), and the Wnt/β-catenin pathway (Yu et al., 2016; Hasan et al., 2017; Chen et al., 2021).

Piperine is a natural alkaloid mostly found in the Piperacea family, mostly in the fruit of *Piper nigrum* (black pepper seeds) and the spikes and roots of *Piper longum*. Piperine has been investigated by several studies that found several therapeutic effects, including anti-oxidant, antimicrobial, anticancer, immunomodulatory, and anti-fibrotic effects (Katarina et al., 2019; Haq et al., 2021). It has shown promising results against fibrosis by reducing cardiac fibrosis through the PPAR-γ pathway (Ma et al., 2017) and pancreatic fibrosis through the TGF-β1/Smad pathway (Choi et al., 2019a). The current study aims to evaluate the potential efficacy of piperine in the treatment of liver fibrosis induced with thioacetamide in mice through the modulation of miR-17 and TGF-/Smad pathways.

## Materials and Methods

### Animals

Male albino mice (15–20 g) purchased from Egyptian Company for the Production of Vaccines, Sera, and Drugs (EGYVAC; Cairo, Egypt) were used in the current study. They were housed in plastic cages under constant conditions of temperature (25 ± 3°C) and humidity (50%) at October University for Modern Sciences and Arts’ animal house. Standard pellet chow (El-Nasr Co., Egypt) and free water were available.

### Drugs and Chemicals

Piperine and thioacetamide were purchased from Sigma-Aldrich (Saint Louis, MO, United States). The other chemicals used were all of analytical grade.

### Induction of Liver Fibrosis

Thioacetamide (150 mg/kg; i.p.) dissolved in saline was injected three times a week for 6 weeks for induction of liver fibrosis. This induction method was chosen based on previous literature (Choi et al., 2019b; Yang et al., 2019).

### Experimental Design

Mice were categorized into three groups (*n* = 6) at random. The first set of mice served as the normal control group. The liver fibrosis control group was injected with thioacetamide (150 mg/kg; i.p.) 3 times/week for 6 weeks. The last group was treated with piperine (10 mg/kg/day; p.o.) for 3 weeks starting from the fourth week of the experiment. The dose and route of administration of piperine were determined based on previous literature (Stojanović-Radić et al., 2019; Choi et al., 2019a).

At the end of the sixth week, blood samples were collected *via* the retro-orbital plexus for serum separation and investigation of the liver enzymes that were analyzed using commercial kits (Biodiagnostic; Cairo, Egypt).

The mice were then terminated *via* cervical dislocation under ether anesthesia, and the livers were quickly dissected out and washed in ice-cold saline. RNA extraction from the isolated livers was used for analysis of the expression of miR-17-5p, Smad-7, Smad-3, collagen-a1, and TGF-β1 through qRT-PCR. Sections from the isolated livers were fixed in formalin and used for the histopathological examination and the investigation of the immunohistochemical activity of TNF-α and α-SMA.

### Quantitative Real-Time Polymerase Chain Reaction

The isolated livers were used for total RNA isolation using Trizol (Invitrogen; Auckland, New Zealand) according to the manufacturer’s instructions and reverse-transcribed into cDNA with the Reverse Transcriptase M-MLV (Promega, Madison, WI, United States).

Primer sequences to be used in the experiment were as follows:

**Table:** 

Gene	Forward primer	Reverse primer
U6	CGC​TTC​GGC​AGC​ACA​TAT​ACT​A	CGC​TTC​ACG​AAT​TTG​CGT​GTC​A
miRNA 17-5p	TCTAGATCCCGAGGACTG	ATCGTGACCTGAA
Col1A1	TAA​CTT​CTG​GAA​TTC​GAC​TTT TTGG	GTC​CAG​CCC​TCA​TCC​TGG​CC
Smad7	CTC​GGA​CAG​CTC​AAT​TCG​GA	CAG​TGT​GGC​GGA​CTT​GAT​GA
Smad3	TGG​CCA​CTG​CTG​CTT​CCT​CTT​CTT	GGGGCC AGCTTCGTCATACTCCT
TGF-β1	CATCCATGACATGAACCG ACCCTT	ACA​GAA​GTT​GGC​ATG​GTA​GCC​CTT

Small RNA species-enriched RNA was isolated based on the manufacturer’s instructions (mirVana miRNA isolation kit; Ambion, Austin, TX, United States) for miRNA quantitative reverse transcriptase PCR. miRNA was reverse-transcribed by using Ncode miRNA first-strand complementary DNA synthesis kits (Invitrogen). Quantitative reverse transcriptase PCR was accomplished using a Power SYBR Green PCR Master Mix on a CFX96 Instrument (Bio-Rad, United States). The relative standard curve method was used for data analysis.

### Histopathologic Assessment of Hepatic Tissue Damage

The isolated livers from all the mice groups were fixed in 10% formalin solution. Liver sections were collected on glass slides, deparaffinized, and stained by hematoxylin & eosin stain for routine histopathological examination using an electric light microscope. This is according to the method previously described by Bancroft and Gamble (Bancroft and Gamble, 2008).

### Immunohistochemical Reaction of TNF-α and α-SMA

Sections from liver tissue of around 3-µm thickness embedded in paraffin were used for detection of TNF-α and α-SMA through the immunostaining with primary antibody polyclonal immunoglobulin-G of mice TNF-α and α-SMA according to the method previously described by Zaafan et al. (2019). Finally, grading of immunohistochemical reactivity from 1 to 3 was performed.

### Statistical Analysis

Data are presented in the form of mean ± SEM. The comparisons among means of different groups were done *via* one-way analysis of variance (ANOVA) and Tukey–Kramer multiple comparisons posttest (Stoline et al., 1981). The Kruskel–Wallis test was used for analyzing the histopathological scores, followed by Dunn’s multiple comparisons test. The level of significance was taken as *p* ˂ 0.05. All the statistical tests were carried out using GraphPad Prism software package, version 5 (GraphPad Software, Inc., United States).

## Results

### Effect of Piperine on Serum Levels of Liver Enzymes in Liver Fibrosis

**FIGURE 1:**
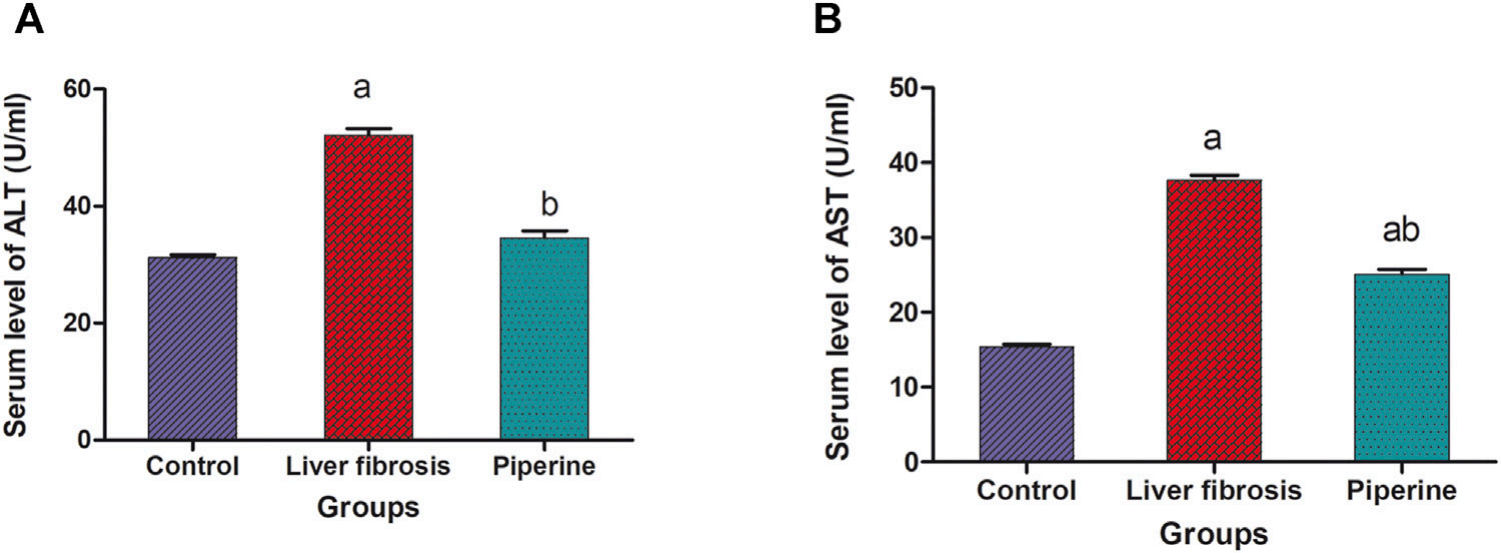
Effect of piperine treatment on the serum levels of the liver enzymes, alanine transaminase (ALT) and aspartate transaminase (AST), in mice with thioacetamide-induced liver fibrosis. The data are presented as mean ± SEM (*n* = 6). a, significant difference from the control group; b, significant difference from the liver fibrosis inducted group (at *p* < 0.05).

The results revealed a significant increase in the serum levels of liver enzymes upon thioacetamide injection. Alanine transaminase (ALT) was increased by 1.7 fold and aspartate transaminase was increased by 2.4 fold in the mice with liver fibrosis compared to the control group. On the other hand, treatment with piperine significantly decreased the serum levels of ALT by 33.6% and AST by 33.4% compared to the liver fibrosis group (Figure 1).

### Effect of Piperine on miR-17-5p/Smad-7/Smad-3 Signaling in Liver Fibrosis

**FIGURE 2:**
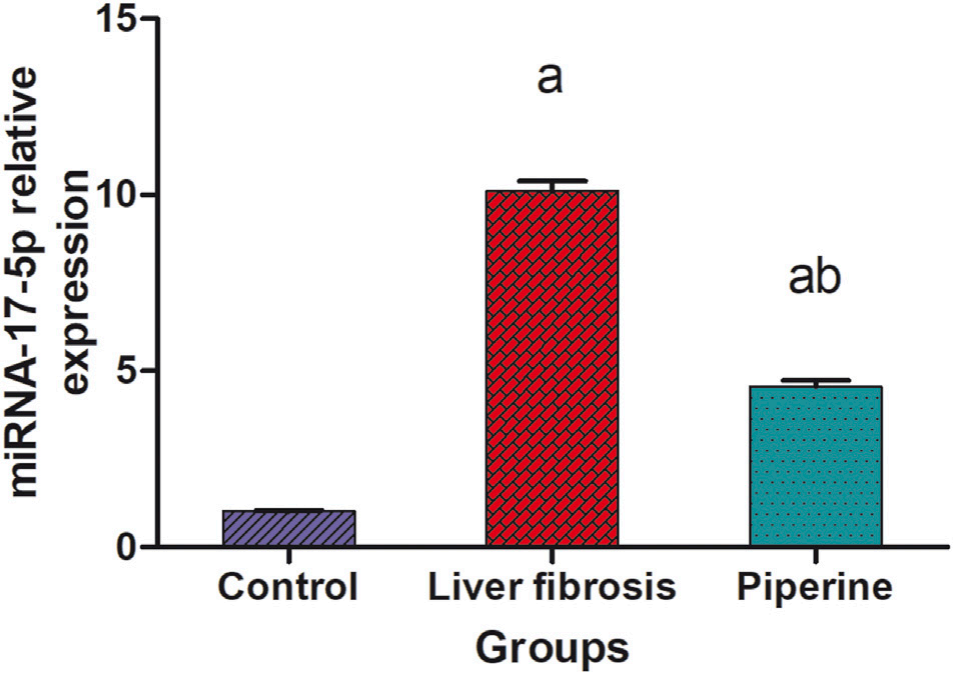
Effect of piperine treatment on the relative expression of miRNA-17-5p in the liver tissue of mice with thioacetamide-induced liver fibrosis. The data are presented as mean ± SEM (*n* = 6). a, significant difference from the control group; b, significant difference from the liver fibrosis inducted group (at *p* < 0.05).

A significant 10.1-fold elevation was observed in the hepatic level of miR-17-5p in the liver fibrosis group compared to the control group. Conversely, treatment with piperine significantly lowered the hepatic level of miR-17-5p by 54.5% compared with the liver fibrosis mice (Figure 2).

**FIGURE 3:**
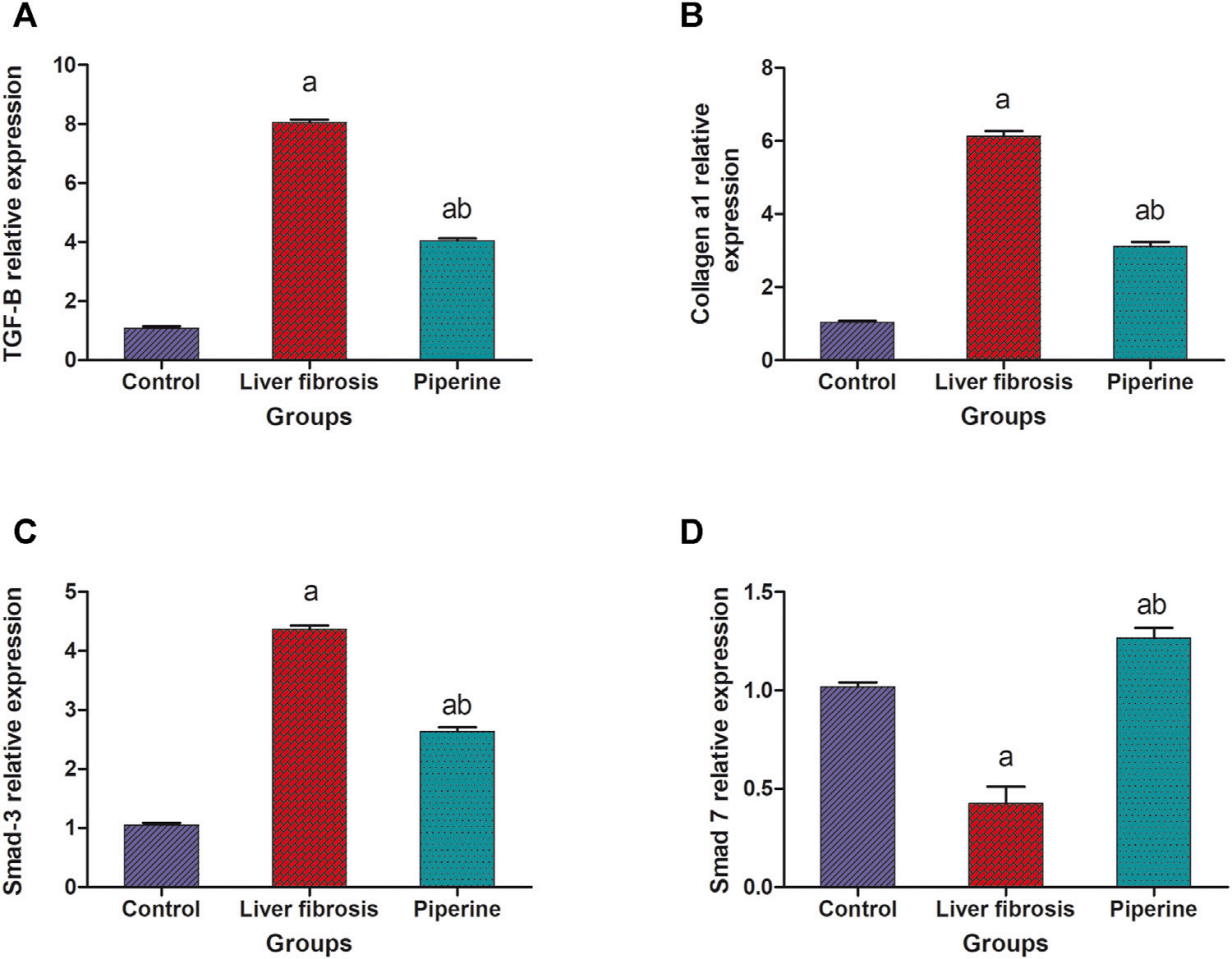
Effect of piperine treatment on the relative expression of **(A)** transforming growth factor-β (TGF-β), **(B)** collagen a1, **(C)** smad-3, and **(D)** smad-7 in the liver tissue of mice with thioacetamide-induced liver fibrosis. The data are presented as mean ± SEM (*n* = 6). a, significant difference from the control group; b, significant difference from the liver fibrosis inducted group (at *p* < 0.05).

The mice with liver fibrosis exhibited a significantly suppressed expression of smad-7 in the liver by 57.0% compared to the control group. This effect was accompanied by a significantly elevated expression of smad-3 and TGF-β1 by 4.3- and 8.1-fold elevation, respectively, compared to the control group. Treatment with piperine abolished these effects successfully and resulted in significantly elevated liver expression level of smad-7 with 3.1 folds compared to the fibrosis group and suppression of the expression levels of smad-3 and TGF-β1 by 40.9 and 49.4% compared to the fibrosis group (Figures 3A,C,D).

### Effect of Piperine on the Hepatic Collagen a1 Expression in Liver Fibrosis

The results represented in Figure 3B showed that the hepatic level of collagen a1 was significantly raised by 6.1 folds in the liver fibrosis group compared to the control group. On the other hand, piperine treatment resulted in significant decline in the hepatic level of collagen a1 by 49.2% compared to the liver fibrosis group.

### Effect of Piperine on Immunohistochemical Reactivity of TNF-α and α-SMA in Liver Fibrosis

**FIGURE 4:**
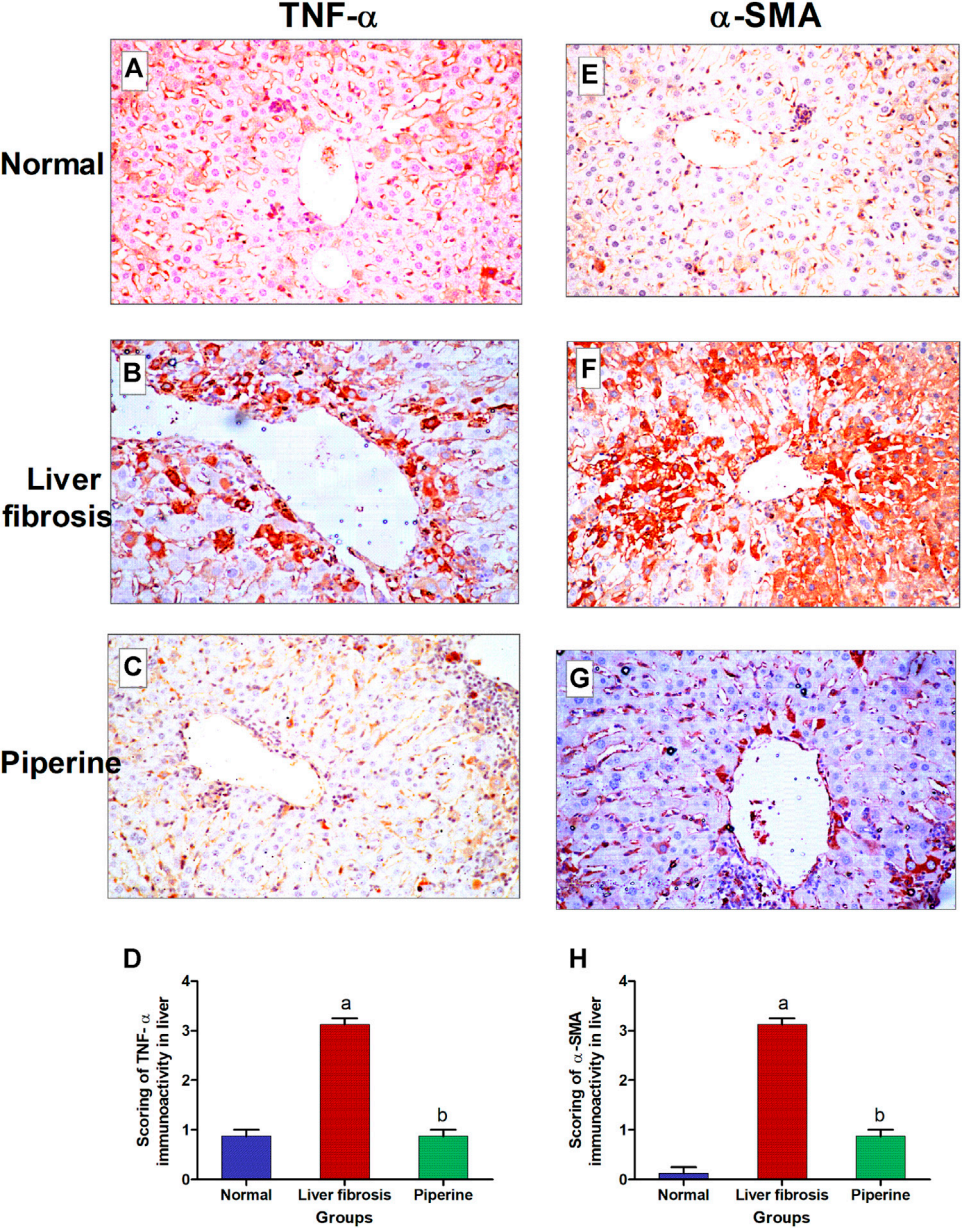
Immunostaining of tumor necrosis factor-α (TNF-α) and α-smooth muscle actin (α-SMA) in the liver tissue of mice with thioacetamide-induced liver fibrosis (H&E×40): **(A)** TNF-α/control group, **(B)** TNF-α/liver fibrosis group, **(C)** TNF-α/dasatinib-treated group, **(E)** α-SMA/control group, **(F)** α-SMA/liver fibrosis group, and **(G)** α-SMA/dasatinib-treated group. **(D, H)** represent a comparative quantification of the immunohistochemical expression for TNF- α and α-SMA in the hepatic tissue of mice from all groups. The severity of the immunoactivity is depending on the intensity and distribution of the brown color. a, represents a significant difference from the normal control group; b, a significant difference from the liver fibrosis inducted group (at *p* < 0.05).

The immunostaining for TNF-α showed weak expression in the hepatic tissue of the normal control mice (Figure 4A). The expression of TNF-α noticeably increased in the hepatocytes surrounding the central vein and the hepatocytes surrounding the portal area upon induction of liver fibrosis (Figure 4B). Piperine treatment resulted in normalization of TNF-α expression in the hepatic tissue (Figure 4C). Liver sections from normal control mice (Figure 4E) exhibited relatively negative expression of α-SMA. Mice with thioacetamide-induced fibrosis showed strong expression of α-SMA in the hepatic parenchyma compared to the control group (Figure 4F). Piperine treatment markedly decreased the expression of α-SMA in the hepatic tissue (Figure 4G) with marked improvement from the inducted group. Comparative quantification of the immunohistochemical expression for α-SMA and TNF- α in hepatic tissue of mice from all groups is presented in Figures 4D,H.

### Effect of Piperine Treatment on Histopathological Alterations of the Liver

**FIGURE 5:**
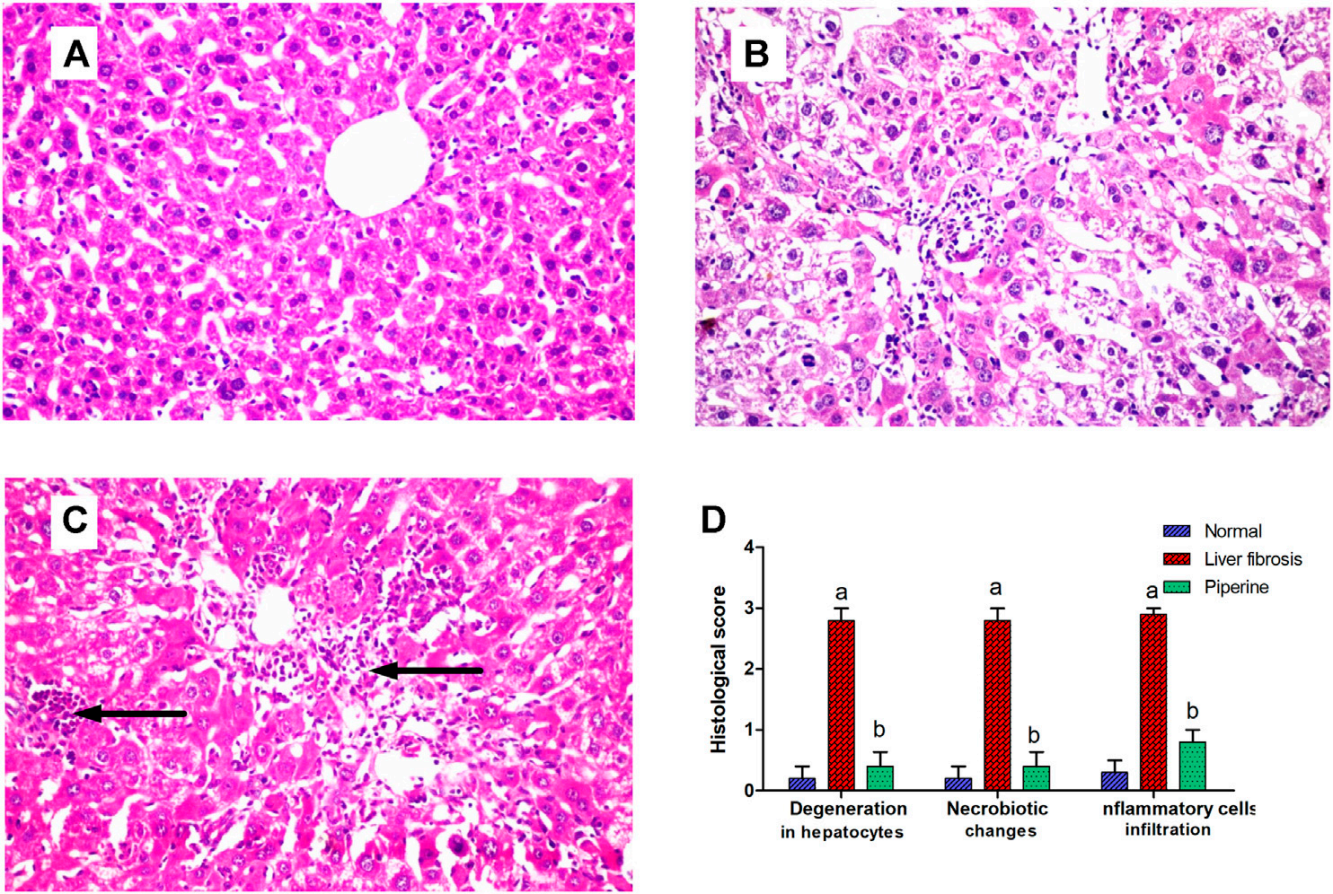
Effect of piperine treatment on the histopathological alterations in the liver tissue in mice with thioacetamide-induced liver fibrosis (H&E×16): **(A)** normal control group, **(B)** liver fibrosis group, **(C)** piperine-treated group, **(D)** scoring of the histological observations in the hepatic tissue from all groups. Data are presented as mean ± SEM of 6 random nonoverlapping fields/section. a, significant difference from the control group; b, significant difference from the liver fibrosis inducted group (at *p* < 0.05).

The liver of the normal mice showed the normal histological structure of hepatocytes surrounding the central vein with no histopathological alteration (Figure 5A). Livers of mice injected with thioacetamide showed degeneration and necrobiotic changes observed in the hepatocytes that were associated with inflammatory cell infiltration in a diffuse manner in between (Figure 5B). Liver sections from the piperine-treated mice showed only inflammatory cell infiltration in the portal area in a focal manner and between the hepatocytes with marked improvement from the liver fibrosis group (Figure 5C). Scoring of the hepatic tissue histological alterations is presented in Figure 5D.

## Discussion

Hepatic fibrosis is a crucial consequent event to chronic liver injury. Piperine’s ability to alleviate thioacetamide-induced liver fibrosis was investigated in this study, as well as the underlying molecular role of miR-17 and TGF-β/Smad pathways.

In the current study, the fibrogenic thioacetamide (TAA) induction model has been prioritized over the carbon tetrachloride (CCl_4_) and common bile duct ligation (CBDL) models. TAA itself is not involved directly in fibrosis, but its metabolites are highly bound to proteins leading to development of oxidative stress (Nevzorova et al., 2020). TAA and CCl_4_ can induce fibrosis, commonly with a delay of progression to necrosis, cirrhosis, and/or carcinoma states. However, TAA has shown a very similar fibrotic environment to that of the liver of humans in addition to long persistence of fibrotic conditions, even after its withdrawal, when compared to other models. This makes it more suitable to investigate relatively long-term therapy for fibrosis. CBDL has been excluded for its common surgical complications such as leakage and mortality rate due to gallbladder rupture (Takahashi and Fukusato, 2017; Nevzorova et al., 2020).

In the present study, piperine showed very promising signs for alleviation of hepatotoxicity resembled in the reduction of liver enzymes, AST and ALT. These results are consistent with those of several other studies where piperine reduced AST and ALT in models of cyanotoxin-induced hepatotoxicity (Abdel-Daim et al., 2019), high-fat diet–induced hepatic steatosis (Choi et al., 2013), and renal ischemia reperfusion–induced liver injury (Mohammadi et al., 2019).

Results of the current study revealed that piperine aided in attenuation of liver fibrosis through manipulation of the TGF-β/Smad signaling pathway *via* restoring of relative expression levels of several pro- and anti-fibrotic molecules. Piperine decreased the expression level of TGF-β in the current study. This is consistent with another study that demonstrated less fibrotic effect of TGF-β on Hep G2 cells by pretreatment of piperine (Marques da Fonseca et al., 2020). Also, piperine has been proven to have the same effect on TGF-β expression through two previously mentioned studies, in cardiac fibrosis (Ma et al., 2017) and pancreatic fibrosis (Choi et al., 2019a).

Consequently, TGF-β reduction affected TGF-β/Smad pathway signaling against fibrosis. That was presented in our study by the decrease in the contributor Smad-3 expression level. The reduction of TGF-β has been extensively proven to affect the expression level of the consequent smad-2 and smad-3 phosphorylation and complexation (Meng et al., 2016; Choi et al., 2019a; Dewidar et al., 2019). Also, the direct role of piperine has been proven to affect Smad signaling in *ex vivo* A549 cells representing human lung adenocarcinoma (Marques da Fonseca et al., 2020) and *in vivo* mouse models of pancreatic fibrosis (Choi et al., 2019a).

Additionally, piperine showed significant reduction of the TNF-α level, which is one of the main cytokines responsible for triggering of fibrosis *via* transformation of HSCs into fibroblasts. This anti-inflammatory effect of piperine through TNF-α reduction has been previously proved in microcystin-induced hepatotoxicity (Abdel-Daim et al., 2019), bleomycin-induced pulmonary fibrosis (Zaafan et al., 2016; Zaafan et al., 2019), isoprenaline-induced myocarditis (Viswanadha et al., 2020; Zaafan et al., 2021), and chronic pancreatitis (Choi et al., 2019a).

Moreover, piperine has decreased ECM deposition resembled in the reduction of relative collagen α1 expression and α-SMA deposition in the piperine-treated group. Our results of a significant protective effect of piperine on collagen relative expression and its consequent accumulation in tissues have been demonstrated in myocardial fibrosis in three different studies (Diwan et al., 2013; Ma et al., 2017; Viswanadha et al., 2020). Also, the hepatoprotective effect of piperine through reduction of α-SMA deposition in tissues was found to be consistent with the aforementioned experiments in pancreatic fibrosis (Choi et al., 2019a) and myocardial fibrosis (Ma et al., 2017).

Meanwhile, it has been demonstrated that collagen expression is directly correlated with miR-17-5p expression but in renal fibrosis and HSC-T6 cell lines (Yu et al., 2015; Fu et al., 2021), which is consistent with our results, since the regression of the pro-fibrotic miR-17-5p and progression of the anti-fibrotic Smad-7 relative expressions have been manifested in the piperine-treated group. These results assure the role of piperine in successful prevention of hepatic fibrosis. Similar results were demonstrated in other studies on myocardial fibrosis (Zhang et al., 2018), CCl_4_-induced model to HSC-T6 cell lines (Yu et al., 2015), and renal fibrosis (Fu et al., 2021), in which miR-17-5p regression has been correlated with fibrosis amelioration *via* upregulation of the protective Smad-7.

Accordingly, we can conclude that piperine possesses a potential therapeutic effect on hepatic fibrosis by restoration of liver enzymes, AST and ALT levels and through targeting the TGF/Smad signaling pathway resembled in the decrease in expression levels of TGF-β, Smad-3, and miR-17-5p and the increase in the expression level of the negative feedback inhibitor Smad-7, in addition to regression of ECM accumulation by reducing α-SMA deposition and collagen-α1 relative expression.

## Data Availability

The original contributions presented in the study are included in the article/supplementary files, further inquiries can be directed to the corresponding author.
